# Diagnosis of latent tuberculosis among HIV infected patients in Ilorin, Nigeria using tuberculin skin test and interferon gamma release assay

**DOI:** 10.11604/pamj.2021.38.24.24039

**Published:** 2021-01-12

**Authors:** Adeniyi Olatunji Aladesanmi, Olutobi Babatope Ojuawo, Olutosin Olukemi Aladesanmi, Ademola Emmanuel Fawibe, Olufemi Olumuyiwa Desalu, Ayotade Boluwatife Ojuawo, Christopher Muyiwa Opeyemi, Mosunmoluwa Obafemi Adio, Victoria Oluwaloniola, Alakija Kazeem Salami

**Affiliations:** 1 Department of Medicine, University of Ilorin Teaching Hospital, Ilorin, Kwara State, Nigeria,; 2 Calderdale and Huddersfield NHS Trust, West Yorkshire, UK,; 3 Department of Paediatrics, University of Ilorin Teaching Hospital, Ilorin, Kwara State, Nigeria,; 4 Department of Medical Microbiology and Parasitology, University of Ilorin Teaching Hospital, Ilorin, Kwara State, Nigeria

**Keywords:** Latent tuberculosis, HIV, Ilorin

## Abstract

**Introduction:**

Latent Tuberculosis Infection (LTBI) screening is recommended for individuals with a known risk factor for progression to active disease especially in the setting of HIV infection. This will ensure early diagnosis and prompt treatment. The purpose of our study was to compare tuberculin skin test (TST) with Interferon Gamma Release Assay (IGRA) in the diagnosis of LTBI among patients with known HIV infection at University of Ilorin Teaching Hospital (UITH), Ilorin.

**Methods:**

this was a hospital based cross-sectional study at the Highly Active Antiretroviral therapy (HAART) Clinic and medical wards of the University of Ilorin Teaching Hospital, Ilorin, Nigeria. A total of 282 consenting patients with HIV infection were recruited. Sociodemographic and clinical information was obtained using a well-structured questionnaire. The screening for LTBI was done using Tuberculin skin test (TST) and Interferon Gamma release assay (IGRA).

**Results:**

the prevalence of LTBI among HIV infected patients was 40.6% and 53.1% using TST and QFT-IT respectively, while the overall prevalence considering positivity to either of the test was 66%. There was mild agreement (κ: 0.218) between TST and QFT-IT in the diagnosis of LTBI among patients with HIV infection. The association between CD4 count and TST was not statistically significant (p value = 0.388) but there was strong association between CD4 cell count and QFT results (p = 0.001).

**Conclusion:**

the prevalence of LTBI is quite high among patients with HIV infection in our locality. There is a need to encourage screening of at-risk individuals to forestall the morbidity and mortality associated with TB in this population.

## Introduction

Tuberculosis (TB) constitutes a serious public health challenge, making the World Health Organization (WHO) declare the disease a situation of world emergency [1]. TB is the most prevalent disease in patients living with the human immunodeficiency virus (HIV) especially in sub-Saharan Africa and it is the major cause of death with a specific aetiology among them [2]. Active TB may result from progression of previously acquired tuberculous infection called latent TB. About one-third of the world's population has LTBI [1]. Persons with LTBI are asymptomatic and non-infectious and the microorganism may persist throughout a person´s life time [3, 4]. There is a 5-10% risk of disease reactivation in immunocompetent persons throughout their lifetime compared to the annual risk among patients living with HIV [1, 4], even if they use antiretroviral chemotherapy [5]. Hence, early diagnosis and treatment of LTBI among HIV infected individual is strongly emphasized [6]. There are currently two diagnostic methods that support the diagnosis of LTBI. These are Tuberculin skin test (TST) and Interferon Gamma Release Assays (IGRAs). Tuberculin skin test (TST) is based on purified protein derivative (PPD) and has been an important tool for detecting LTBI for almost a century [7]. The test however has some limitations which include reader´s variability and false positive test result due to cross reactivity with environmental non-tuberculous mycobacterium and Bacillus Calmette - Guérin vaccine (BCG). Other limitations are false negative test result due to anergy in immunosuppressed individuals and inconvenience to patients who are required to return after 48 -72 hours to get the test read.

The newer diagnostic test for LTBI, the T-cell based Interferon Gamma Release Assays (IGRAs) have been developed. Two types are commercially available, one is based on the ELISPOT (T-spot TB) while the other utilizes the enzyme linked immunosorbent assays techniques (Quantiferon TB - Gold in tube). They are both based on the principle that T-cells of an individual who has acquired TB infection will respond by secreting Interferon gamma (IFN-γ) when re-stimulated by MTB antigen like the early secretory antigenic target-6 (ESAT-6), culture filtrate protein 10 (CFP-10) and the TB7.7 antigens [8]. Unlike the TST, neither BCG vaccine nor infection by non-tuberculous Mycobacteria interferes with IGRA tests, though IGRAs are more costly and technically complex than TST [9]. There are few data on the performance of IGRAs in low and middle-income countries despite the high TB and/or HIV burden in these countries. LTBI screening is recommended for those with a known risk factor for progression to active disease, such as immunosuppression especially HIV infected patients so as to diagnose and commence treatment early. There is however dearth of information in our locality regarding LTBI in HIV infected individuals. This study therefore aimed at determining the prevalence of LTBI among HIV infected persons in a high burden area like our country. This is in line with the main tasks of the Stop TB partnership project that targets controlling the worldwide impact of TB through more rapid detection and treatment of LTBI, particularly in HIV infected patients [10].

## Methods

**Study design and location:** this research work was a hospital based cross-sectional study conducted at the Highly Active Antiretroviral Therapy (HAART) Clinic and medical wards of the University of Ilorin Teaching Hospital, Ilorin, Nigeria.

**Study subjects:** adult patients, 18 years and above who had been previously diagnosed with HIV infection were recruited over a 6-month period from September 2018 to February 2019. Simple random sampling method was used to recruit patients from the HAART clinic and medical wards. The following patients were however excluded: a) patients with features suggestive of active Tuberculosis according to WHO initial active TB screening tool [11]; b) patients who have been treated for active TB in the past; c) patients on intermittent preventive therapy (IPT) for TB; d) patient who did not consent to return within 48-72 hours for reading of the TST.

**Procedures and instruments of data collection:** a well-structured questionnaire was administered by the researchers to obtain each patient´s demographics such as the age, sex, occupation etc. Relevant history to evaluate symptoms of active TB such as cough, hemoptysis, weight loss, fever, drenching night sweat, difficulty in breathing and chest pain was documented as well as risk factors for tuberculous disease in patients with HIV infection. These risk factors included contact with an index case, past treatment for TB and other factors that influence immune status such as alcohol abuse, diabetes etc. The latest CD4 cell counts within the preceding six months were also obtained from the patient´s records and documented. The screening for LTBI was done using the two available diagnostic test - TST (Mantoux technique) and IGRA (QFT- GIT). The tuberculin skin test (TST) was performed using the Mantoux technique. A 0.1mL (5 tuberculin units, produced by BB-NCIPD) intradermal injection of purified protein derivative (PPD) was done on the volar surface of the forearm till an initial wheal of 6 to 10 mm in diameter was raised. Reaction size was determined after 72 hours. When interpreting the TST result, the transverse diameter of induration and not the erythema, was measured in millimeters. The measurement across the forearm was made perpendicular to the long axis following standardization, using the ball-point pen and ruler method to limit reader´s variability. An induration greater than 5mm was regarded as a positive TST in the subjects while induration less than 5mm was taken as a negative test.

The QuantiFERON-TB GOLD in tube (QFT-GIT) by QIAGEN, Thermo Fisher Scientific incorporated was used for the assessment of IGRA. It was performed on fresh venous blood specimens obtained from consenting patients with HIV/AIDS. About 1 ml of blood was obtained directly into one of each of the four manufacturer- precoated, heparinized Quantiferon tubes; the tubes consisted of the Nil control (Grey cap), TB Antigen 1 (Red cap), TB Antigen 2 (yellow) and Mitogen control (purple cap); the vacutainer tubes were made to resist blood flowing into them once they have reached a volume of 1ml. The tubes were shaken about 10 times to ensure that the blood properly mixed with entire content of the tube. They were properly labeled with a number which was linked to the form leading to the identification of patients prior to the blood drawing. The blood samples were transported to the laboratory at room temperature within the recommended 16 hours of collection by the manufacturer for processing. The tubes were incubated upright at 37°C for 21 hours (overnight) and centrifuged for 15 minutes at 3000 RPM. Re-centrifugation was considered at higher speed if there was no proper separation into a supernatant. The plasma harvested after centrifugation was adequately stored at -30°C before assessment of the concentration for IFN-γ, which was carried out using ELISA test. Samples with ≥0.35 IU/mL IFN-γ following stimulation with *M. tuberculosis* specific antigens were considered positive, while samples with < 0.35 IU/mL were considered negative. The QFT-IT test result were considered indeterminate if production of IFN-γ after stimulation with Mitogen was < 0.8 IU/mL or if the test tube optical density was < 0.35 IU/mL or < 25% of that of the negative control tube. Both test kits were administered on 10 apparently healthy subjects, who were HIV negative to ensure the validity of the kits before its use in this study. Individuals who were immune reactive to TST or IGRA underwent further evaluation to exclude an active tuberculosis by performing a plain chest radiographs (posterior anterior view) and sputum analysis f^2^or GeneXpert study for those who could expectorate sputum and those with features of active TB were excluded from this study.

**Data analysis:** all data obtained were analyzed using the Statistical package for social science, IBM SPSS statistics® 2012 version 21. Demographic and clinical data of cases were summarized using frequencies, percentages, and proportions. Normally distributed quantitative variables were expressed as mean, standard deviation while median, range and interquartile range (IQR) were used for variables not normally distributed. Qualitative variables were compared using Chi-square tests, while quantitative variables were compared using Independent sample T test as appropriate. The strength of agreement of between TST and IGRA test was assessed using Cohen kappa coefficient which measures inter-rater agreement for qualitative variables. Statistical significance was set at p value < 0.05.

**Ethical approval:** it was obtained from the Ethical Review Committee of the University of Ilorin Teaching Hospital. An informed consent was obtained from every subject using a consent form which stated in clear and simple terms the purpose of the study and the procedures the subject was required to undertake after consenting to participate in the study.

## Results

**Socio-demographic and clinical characteristics of the participants:** a total of 288 subjects were recruited into this study after excluding 14 subjects on account of diagnosis of active TB following further evaluation. The mean age of subjects was 43.1 ± 9.8 years. Majority of the subjects (71.9%) were females with a male to female ratio was 1: 2.5. (Table 1). The duration of diagnosis of HIV infection among the participant ranged from 1 to 15 years, with a mean duration of 5.6 ± 3.6 years. All subjects were on antiretroviral medication. Majority of the subjects 151(52.4%) had their CD4+ counts ≥ 500 cells/μL, 62 (21.5%) had CD4 counts between 351-499 cells/μL, 45 (15.6 %) had a CD4+ count between 200 - 350 cells/μL while only 30(10.4%) had their CD4+ cell count less than 200 cell/μL. The median CD4+ cell count of the subjects was 525 cells/μL with an inter-quartile range (IQR) between 342.5 - 732.8 cells/μL (Table 1).

**Table 1:** socio-demographic and clinical variables of the study participants

Variable	Frequency (%)
**Age group**	
≤ 30	24 (8.3)
31 - 40	108 (37.5)
41 - 50	108 (37.5)
51 - 60	27 (9.4)
61 - 70	18 (6.3)
> 70	3 (1.0)
Mean ± SD (43.07 ± 9.81) Range: 23-75	
**Sex**	
Male	81 (28.1)
Female	207 (71.9)
**Level of Education**	
No formal education	51 (17.7)
Primary	45 (15.6)
Secondary	69 (24.0)
Tertiary	123 (42.7)
**Marital status**	
Single	30(10.4)
Married	231(80.2)
Divorced	9 (3.1)
Widowed	12(4.2)
Separated	6 (2.1)
**CD4 Count Levels (cells/μL)**	
< 200	30 (10.4)
200 - 350	45 (15.6)
351 - 499	62 (21.5)
>500	151 (52.4)
Median CD4 Count	525.0

**Prevalence of latent tuberculous infection among HIV Patients in UITH:** it was obtained using the two available tests. Considering Interferon gamma release assay (QFT-GIT), 53.1% of the participants had a positive reaction. On the other hand, using Tuberculin skin test (Mantoux technique) - 40.6% had a positive reaction. Seventy- eight (78) subject had positive reaction to both test while seventy -five (75) subjects reacted positively only to QFT-GIT and Thirty-nine (39) reacted positively only to TST. The total number of subjects with a positive LTBI result to either test was One hundred and ninety-two (192). Hence, the overall prevalence of LTBI among the subjects from this study when considering positivity to both QFT-GIT and TST or either of them was 66.7%.

**Strength of agreement of tuberculin skin test and Quantiferon TB gold in tube:** the strength of agreement of tuberculin skin test and Quantiferon TB gold in tube in the diagnosis of LTBI was determined using Cohen kappa´s measure of agreement. This study found a fair degree of agreement between Quantiferon and TST (k: 0.218) which was statistically significant (p value < 0.001). Two thirds of the subjects had a positive reaction to both TST and Quantiferon TB test (Table 2).

**Table 2:** the strength of agreement between TST and IGRA in the diagnosis of LTBI in HIV infected patients

	TST				
	Positive	Negative	Total	K	p-value
**Quantiferon**	n (%)	n (%)	n (%)		
**Positive**	78 (66.7)	75 (43.9)	153 (53.1)	0.218	< 0.001*
**Negative**	39 (33.3)	96 (56.1)	135 (46.9)		
**Total**	117 (100.0)	171 (100.0)	288 (100.0)		

K: Kappa (Measure of Agreement). *p value <0.05

**Strength of agreement of tuberculin skin test and Quantiferon TB gold in tube across various CD4 count levels:** the strength of agreement across various CD4 cell count is shown on Table 3. There was slight agreement for CD4 count less than 200 cells/μL, with Cohen kappa coefficient of k: 0.048, though this was not statistically significant, (p value 0.794). Between CD4 counts of 200- 350 cells/μL, there was moderate agreement which was significant statistically with a Cohen Kappa coefficient of k: 0.44 (p value = 0.003). Also, between 351 -499 cells/μL, the agreement between the tests was slight with a Cohen Kappa coefficient of k: 0.125 (p value= 0.321). Also, for subjects with CD4 cell count ≥ 500 cells/μL, the agreement was slight and statistically significant with Cohen Kappa´s coefficient of k: 0.216 (p value = 0.002). Therefore, the agreement between both QFT-GIT and TST was more between the CD4 cell count of 200 - 350 cells/μL and least with CD4 count < 200 cells/μL.

**Table 3:** the strength of agreement between TST and IGRA in the diagnosis of LTBI in HIV infected patients based on CD4 count values

	TST Positive	Negative	Total	K	p value
Variable	n (%)	n (%)	N (%)		
**CD4 <200 Quantiferon**					
Positive	3 (33.3)	6 (28.6)	9 (30.0)	0.048	0.794
Negative	6 (66.7)	15 (71.4)	21 (70.0)		
Total	9 (100.0)	21 (100.0)	30 (100.0)		
**CD4200-350 Quantiferon**					
Positive	12 (66.7)	6 (33.3)	18 (40.0)	0.444	0.003*
Negative	6 (33.3)	21 (66.7)	27 (60.0)		
Total	18 (100.0)	27 (100.0)	45		
**CD4 351 - 499 Quantiferon**					
Positive	15 (50.0)	12 (37.5)	27 (43.5)	0.125	0.321
Negative	15 (50.0)	20 (62.5)	35 (56.5)		
Total	30 (100.0)	32 (100.0)	62 (100.0)		
**CD4 ≥ 500 Quantiferon**					
Positive	48 (80.0)	51 (56.0)	99 (65.6)	0.216	0.002*
Negative	12 (20.0)	40 (44.0)	52 934.4)		
Total	60 (100.0)	91 (100.0)	151(100.0)		

**Relationship between diagnosis of LTBI using TST/QFT-GIT and various levels of CD4 cell count:** the association between CD4+ count and LTBI diagnosis using TST was not statistically significant both at the level of Chi square analysis and the Point biserial correlation (p value 0.388 and 0.627 respectively) but the association between CD4 cell count and LTBI diagnosis using QFT-GIT was positive which was statistically significant using both Chi square and Point biserial correlation (p value = 0.001 and < 0.001).

## Discussion

Majority of the study participants were within the ages of 31 and 50 years which corresponds to the reproductive age with the highest burden of HIV infection [12]. Over three quarters of the people were married perhaps due to the fact that single individuals may be more sensitive to stigmatization and avoid screening tests and those who know their status may not seek care early due to denial of reality of their HIV status [13]. The prevalence of LTBI among HIV infected patients in UITH in our study using TST and Quantiferon TB gold in tube were 40.6% and 53.1% respectively but overall prevalence when positivity to any of the two tests were considered was 66%. The prevalence of LTBI by TST is lower than the 55% and 76% values obtained in a previous study done in Zambia [14] and Togo [15]. Additionally, the prevalence of LTBI by QFT-GIT is higher than 42% obtained in Kenya and 47% reported in Brazil [16]. However, contrary to most reports, the QFT-GIT prevalence was higher in this study than TST which is known to be affected by immunization status of individuals as well as infection with many other Non-tuberculous Mycobacterium. This is probably because QFT-GIT has better performance in immune suppressed individuals [17]. When the two test tools were combined as done in some previous studies [18, 19] the overall prevalence of LTBI of 66% as observed in this study was higher than what was obtained in Denmark, the United kingdom and Brazil with prevalence of 4.6%, 10% and 18% respectively probably due to the fact that TB burden is lower in the developed countries than developing countries where this study was done and the highest burden of TB worldwide is in sub-Saharan Africa as well as in Indian subcontinent [19-21]. The prevalence of LTBI from this study was also close to that obtained from a review by Kawatsu *et al*. [22] on LTBI among prisoners in Nigeria where an overall prevalence of 73.0% was obtained. Though a larger study will be required to determine the prevalence of LTBI in Nigeria, our findings may reflect the general prevalence of LTBI in a high TB burden country like Nigeria irrespective of the HIV status. However, this high prevalence is quite alarming and requires prompt preventive measures especially in the setting of HIV where the rate of progression is rapid [23].

A study done in Nigeria to determine prevalence of LTBI among health care workers by Umoh *et al*. [21] in Akwa Ibom reported a prevalence of LTBI of 24.8% and 45.8% as assessed by QFTGIT and TST, respectively. However, among the health worker older than 50 years who may be more at risk due to prolonged contact the LTBI prevalence was 51.3% slightly lower than the prevalence from this study. Also, the study reported a higher prevalence using TST contrary to the finding in this study where QFT-GIT had higher prevalence, the reason for this could not be ascertained but may be due to immunization with BCG or other Non-tuberculous Mycobacterium which causes false positive TST [24]. This study showed that the overall degree of agreement between tuberculin skin test and Quantiferon TB gold in tube in the diagnosis of LTBI was fair. This is in conformity with a previous study by Gislene *et al*. in Brazil among HIV patients [20], which also showed a fair correlation between TST and Quantiferon TB gold in tube. The study by Naashaj *et al*. [15] in Zambia also showed a moderate correlation between the two tests with a coefficient of agreement of 0.53. This was higher than our finding despite the fact that both studies were conducted in the high TB burden countries probably due to the fact that the patients recruited in their study were those with high CD4+ cell count [15]. This agreement found between both tests from this study strengthens the fact that they could be used in place of each other in the diagnosis of latent tuberculosis. Several studies done earlier showed poor correlation between both tests in evaluating patients for LTBI [15], more studies done recently are reporting better agreement between the two tests as we have in this study [15, 20]. Using both tests together for high risk populations like HIV patients will increase the yield of diagnosis of LTBI as demonstrated in this study and prevent disease progression. The prevalence of LTBI was 40.6% with TST alone and 53.1% with QFT but it increased to 66.7% when we considered positivity to either of both tests. This is similar to reports from some other studies, hence concomitant use of both may be necessary [15].

Considering agreement of both test across various CD4+ level this study also found that at CD4+ cell count < 200 cells/μL had no agreement as compared to higher CD4+ counts with the best agreement noticed among those with CD4+ count ranging between 200 and 350 cells/μL. This was similar to the finding by Naashaj *et al*. [15] where CD4+ count greater than 388 cells/μL had better correlation. This differed slightly from what was reported by Inger Brock *et al*. [19] where there was no correlation with CD4+ less than 200 cells/μL but was in contrast with Gislene *et al*. [20] in Brazil where low CD4+ count affected only TST but had no effect on QFT. Therefore, the findings from this study suggest that both tests perform best at higher CD4 count due to anergy. The relationship between degree of immunosuppression and diagnosis of LTBI has had varying reports from previous studies despite the fact that it has been well reported that the lower the CD4+ count, the higher the risk of PLWHA being infected with TB and the greater the rate of progression to active disease [25]. This study found reactivity for both TST and QFT across the various CD4 cell count ranges, although majority (89.6%) of the study participant had CD4+ cell count >200 cells/μL with a mean CD4+ count of 525 cells/μL for all the subjects. The diagnosis of LTBI was more among subjects with higher CD4+ cell count proportionately when compared to those with CD4 count less than 200 cell/μL irrespective of the fact that more people had their CD4+ cell count greater 200 cell/μL among the subjects. This association between CD4+ count and TST was not statistically significant unlike QFT-GIT, hence the performance may be not be affected by CD4+ count value. Some studies have reported that TST and QFT-GIT had sub-optimal performance in immunosuppressed individuals [15, 19], the study in Denmark by Inger Brock *et al*. [19] found a strong correlation between CD4+ cell count level and interferon gamma production, those with CD4+ count less than 100cells/μL had higher proportion of indeterminate results as compared with subjects with CD4+ count greater than 300 cells/μL (24% vs. 2.8%) this was in contrast to our study where we had no indeterminate result irrespective of CD4+ count.

However, in agreement with the Zambian study by Naasha *et al*. [15], that reported better response among subjects with a CD4+ count ≥ 388 cells/ μL for both TST and QFT test this study found a similar result with better response at CD4+ count greater than 351 cells/μL, hence a better performance more likely among early HIV infection. Also, the study by Gislene *et al*. [20] in Brazil reported that TST was only positive in individuals with CD4+ count greater than 300 cells/μL but this was not the case with QFT where reaction was seen among subjects with lower CD4+ cell count in that study but this study also differ from this finding with TST reaction at CD4+ count less than 200 cells/μL. Although, TST may not be as reliable as QFT among those with lower CD4+ count, more studies may be required to determine the cut off CD4+ cell count value where the performance of both test will be considered optimal for evaluating patients for LTBI. Our research is not without a limitation. This hospital-based study was done among HIV seropositive individuals who are under medical treatment and with a good clinical characteristic such as high CD4+ cell count and good compliance to medication. Hence, it may not be entirely representative of the wider community where patient have lower CD4+ cell count, late presentation, and many complications.

## Conclusion

The prevalence of LTBI in HIV positive individuals in UITH is 40.6% and 53.1% using TST and QFT-IT respectively, while the overall prevalence considering positivity to either of the test is 66.7%. This is quite high if we consider the risk it portends to the subjects. There was fair agreement between TST and QFT-GIT in the diagnosis of LTBI among HIV infected individuals which was more marked among those with CD4+ counts above 200 cell/μL showing a positive relationship between the diagnosis of LTBI and the CD4+ cell count of subjects. Routine LTBI screening is therefore advocated among HIV patients who are highly vulnerable. Using both test tools concurrently will increase the yield but if only one of the test is to be done I would recommend QFT-GIT which had a better yield based on this study.

### What is known about this topic

- Active tuberculosis is the leading cause of death among HIV infected individual and is usually a progression of latent tuberculosis;
- Both TST and IGRA are useful tools in the diagnosis of LTBI, the TST has been available for more than 100 years was the only test available for the diagnosis of LTBI but recently, IGRAs were introduced;
- Some studies have shown that IGRA (QTF-G and TB-spot) shows higher specificity than TST especially among BCG vaccinated populations.

### What this study adds

- This study found that the prevalence of LTBI among HIV infected individuals is high;
- It also evaluated the performance and strength of agreement of both test tools in the diagnosing LTBI in a developing country with high TB and HIV burden;
- This study highlighted one of the goals of Stop-TB program which is the importance of early diagnosis of TB at latent phase to enable prompt treatment and prevent active disease.
